# Supplementary material for: First description of a widespread Mytilus trossulus-derived bivalve transmissible cancer lineage in M. trossulus itself
Source: Sci Rep. 2021 Mar 11;11:5809. doi: 10.1038/s41598-021-85098-5 (PMC7970980; doi:10.1038/s41598-021-85098-5)
Supplement: Supplementary file 7 — Supplementary Information. [file 41598_2021_85098_MOESM7_ESM.pdf]

Supplementary Figures and Tables

First description of a widespread *Mytilus trossulus*-derived bivalve transmissible cancer lineage in *M. trossulus* itself

Maria Skazina[1]*, Nelly Odintsova[2], Maria Maiorova[2], Angelina Ivanova[1],
Risto Väinölä[3], Petr Strelkov[1]

[1]Saint-Petersburg State University, Saint-Petersburg 199178, Russia

[2]National Scientific Center of Marine Biology of the Far East Branch of the Russian Academy of Sciences, Vladivostok 690041, Russia

[3]Finnish Museum of Natural History, University of Helsinki, Helsinki P. O. Box 17, FI-00014, Finland

*Corresponding author m.skazina@spbu.ru

Supplementary figures

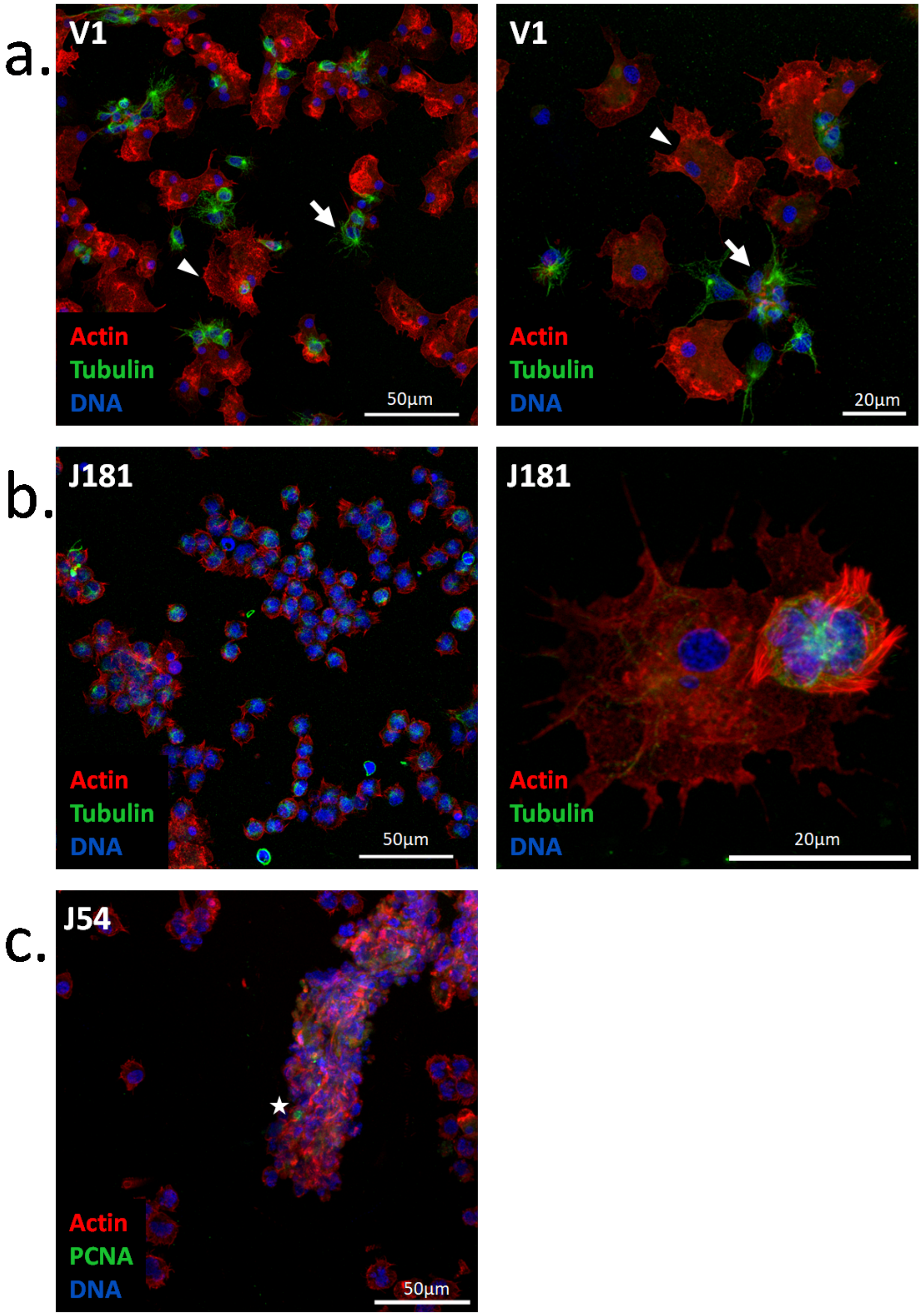

Figure S1. *Mytilus trossulus* hemocytes cultivated for 24 hours. Cells were stained with TRITC-labeled phalloidin (red), and the nuclei (chromatin) were stained by DAPI (blue). Hemocytes of a healthy individual V1 (**a**) and a diseased individual J181 (**b**) were stained with tubulin primary antibodies, hemocytes of a diseased individual J54 (**c**) were stained with PCNA antibodies (green). Arrows in pictures of V1 point to actively moving cells, while arrowheads point to adherent cells. Star in picture of J54 marks the cell positive for proliferation.

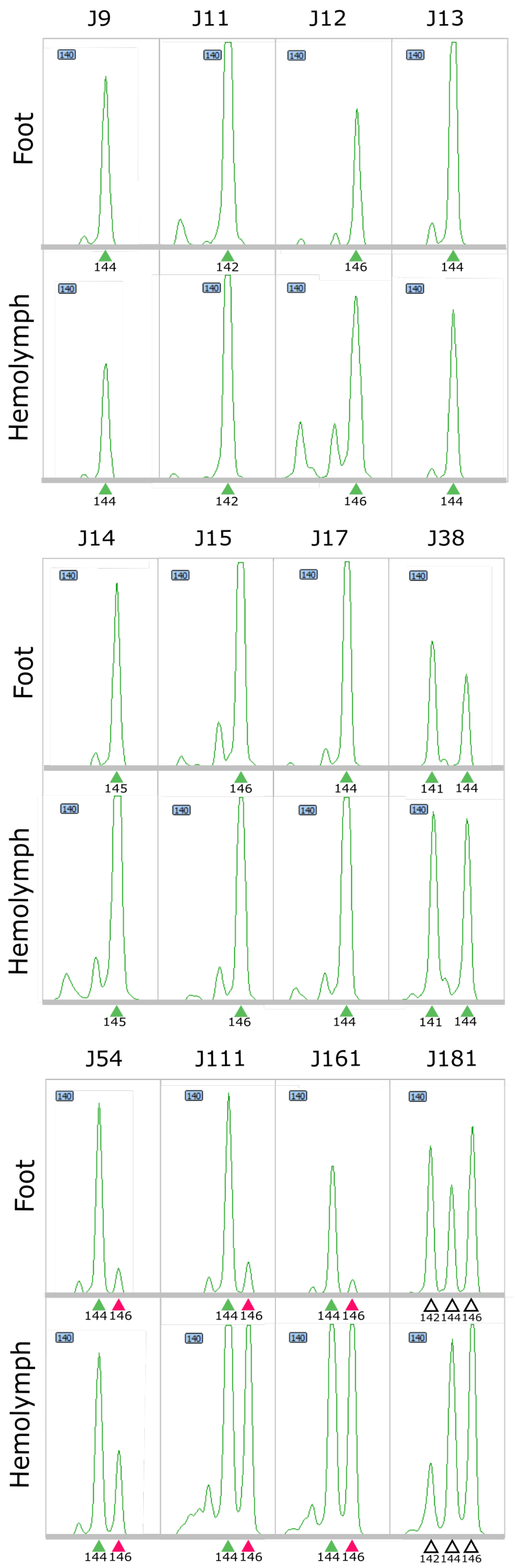

Figure S2. Fragment analysis of *Mg*µ3 microsatellite locus from the hemolymph and the foot tissues of eight healthy mussels (control) and four DN-suggested mussels. Colored triangles designate putatively host-derived *Mg*µ3 fragments (green), putatively cancer-derived *Mg*µ3 fragments (violet); open triangles mark unrecognized fragments. The patterns of *Mg*µ3 fragments in the hemolymph and the foot tissues coincide in healthy mussels; an additional peak is present in the hemolymph of DN-suggested mussels.

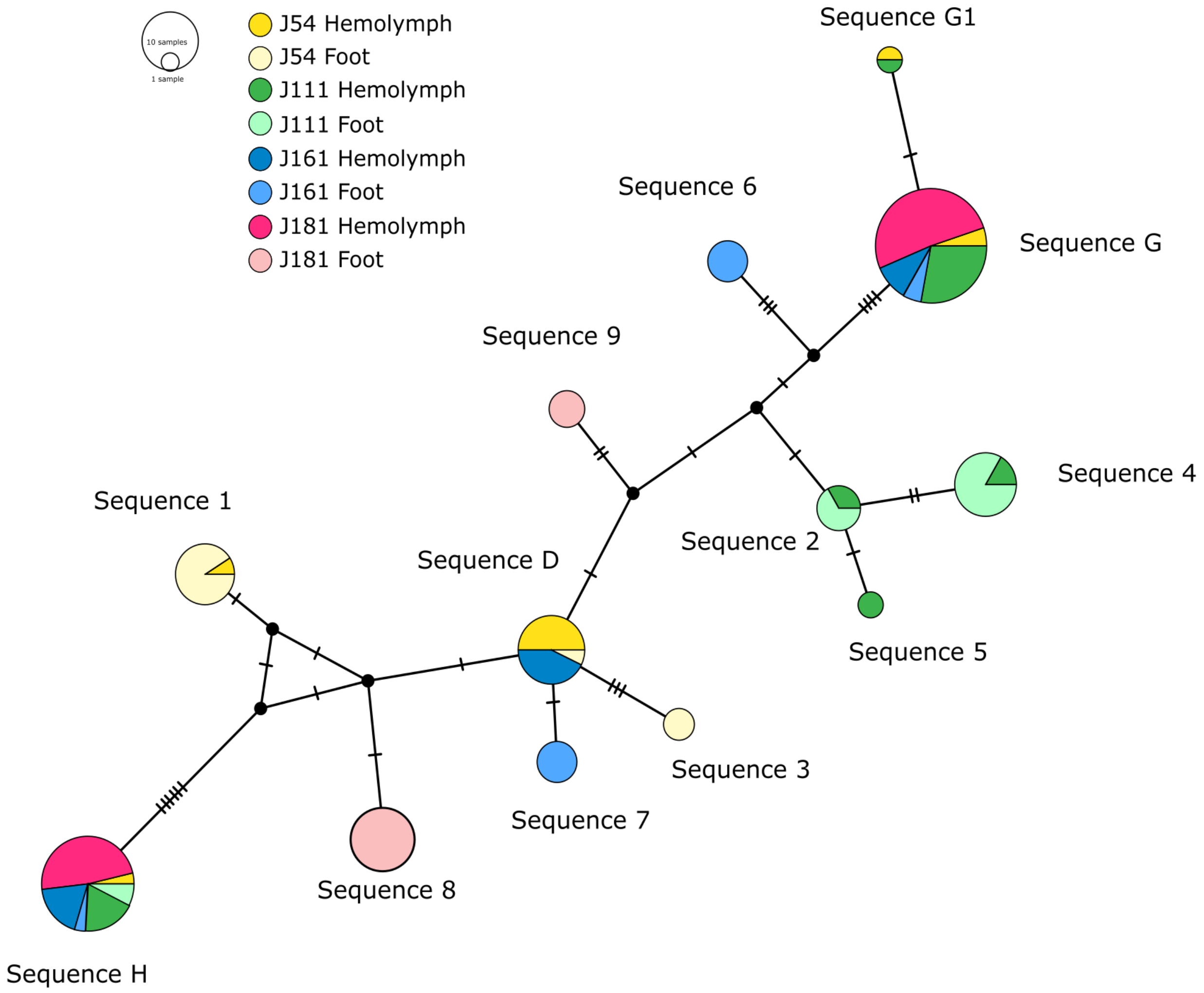

Figure S3. TCS network representing EF1α sequences obtained by molecular cloning. Sequences from individual mussels and from different tissues (hemolymph and foot) are color-coded (see legend on the top). All minor sequences represented by a single bacterial colony have been removed.

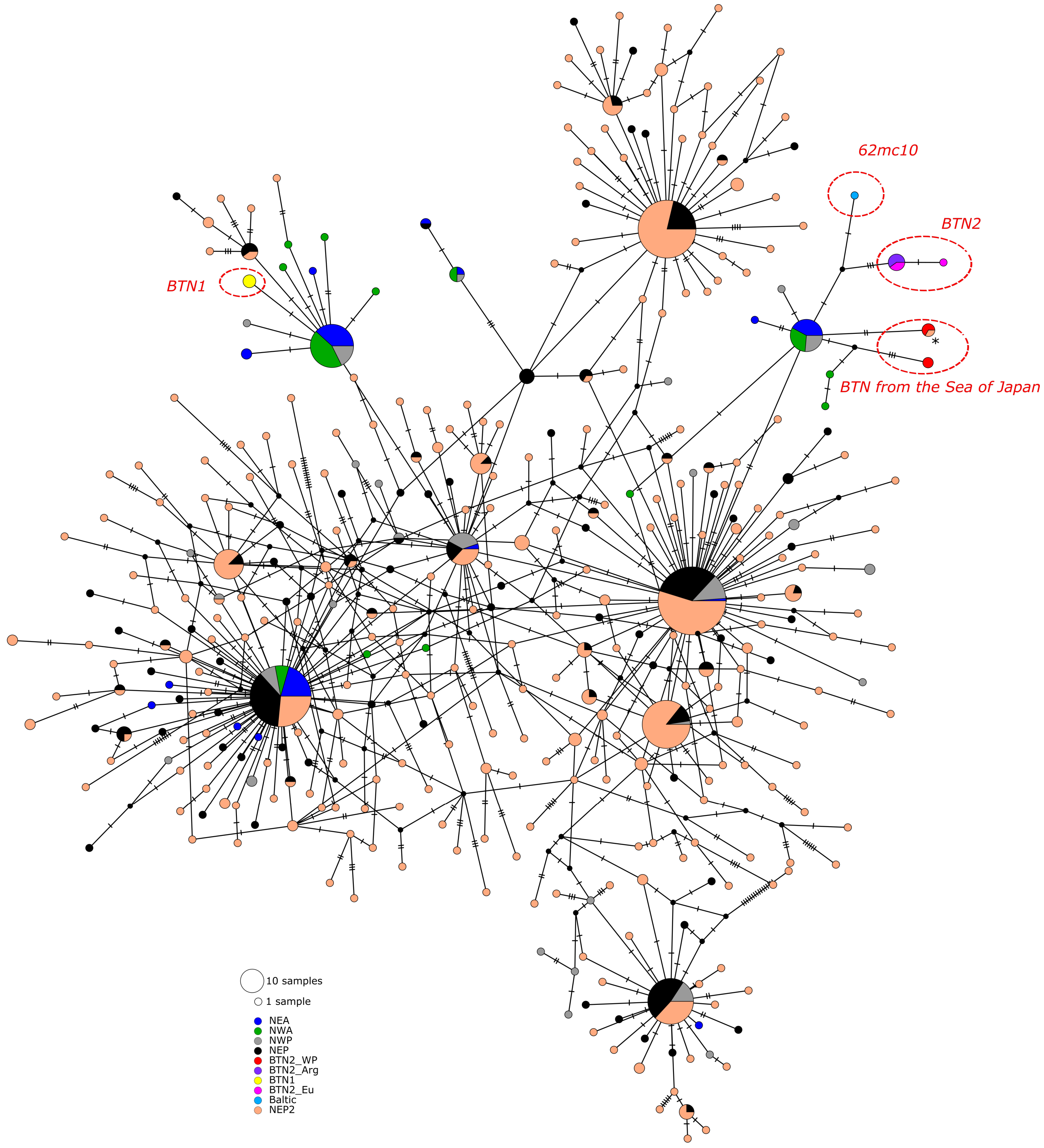

Figure S4. *M. trossulus* and BTN COI haplotype network from the TCS analysis of 542-bp alignment of 843 sequences. The data come from a reanalysis of the results (Fig. 5) with additional 493 sequences from Crego-Prieto *et al.*[54]. Each circle represents a single allele. The size of the circle is proportional to the number of individuals found to bear the allele. Bars indicate mutations between alleles. Small black circles indicate hypothetical haplotypes predicted by the model. The alignment is available as Supplementary Data S5. The geographical origin of samples is color-coded. Samples corresponding to BTN1 (British Columbia) are in yellow, to BTN2 in pink (Europe, BTN2_Eu) and purple (Argentina, BTN2_Arg), to BTN from the SOJ in red (BTN2_WP). Reference *M. trossulus* samples from the Northwest Pacific (NWP) are in grey, from Northeast Pacific (NEP), in black, from Northwest Atlantic (NWA), in green, from Northeast Atlantic (NEA), in dark blue, and from the Baltic Sea (a single sample 62mc10), in light blue (Baltic). The data from Northeast Pacific from Crego-Prieto et al.[54] are in orange (NEP2). The asterisk mark the putatively cancer sequence (NCBI accession number KF931805) from Crego-Prieto et al.[54]

a.

	D alleles, p-value	C alleles, p-value
RDP	-	-
GENECONV	$4.668*10^{-5}$	-
Bootscan	$5.704*10^{-3}$	-
Maxchi	$1.556*10^{-9}$	$4.229*10^{-2}$
Chimaera	$1.145*10^{-4}$	-
SiScan	$6.138*10^{-11}$	$1.248*10^{-17}$
3Seq	$1.042*10^{-14}$	$9.983*10^{-6}$

b.

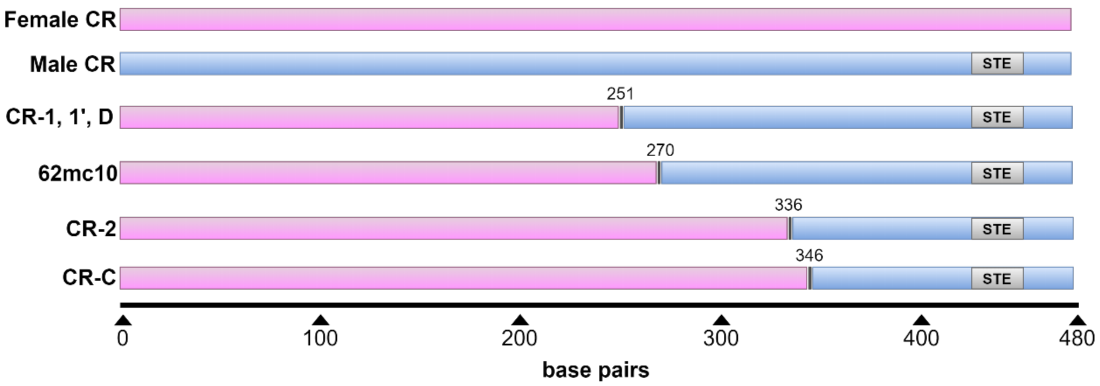

Figure S5. (**a**) 479 bp long alignment subjected to recombination essay in RDP4 package with default parameters included CR alleles of cancerous mussels detected in this study and in the study of Yonemitsu *et al*. (2019), CR of 62mc10 individual from the Baltic Sea and the set of reference male and female CR (see Accession numbers in the alignment included as Supplementary Data S6). The RDP4 package detected two recombination events in the region around 249-263 nucleotide position of alignment in CR-D, D1, D2, D3, 1, 1' and 62mc10 and in the region of 333-360 nucleotide position in CR-C, C1 and 2. RDP4 output p-values (cutoff of 0.05) are represented in the table. (**b**) The putative breakpoints marked at schematically depicted alignment of CR used in the recombination analysis. Each bar represents a sequence. Pink color designates standard F-mtDNA, blue color, standard M-mtDNA. The putative breakpoints are marked with black vertical lines, designated by the nucleotide position from the start of alignment. Sperm transmissible element is marked with a grey box.

Supplementary tables

Table S1. The list of *Mytilus trossulus* and BTN COI sequences used for creating TCS haplotype networks.

NCBI Accession numbers	N of sequences	Source
AY823625	1	Breton et al., 2006
KF643248, KF643275, KF643297, KF643331, KF643385, KF643387, KF643419, KF643426, KF643427, KF643460, KF643487, KF643497, KF643502, KF643600, KF643628, KF643676, KF643701, KF643735, KF643750, KF643757, KF643770, KF643789, KF643795, KF643812, KF643820, KF643889, KF643915, KF643952, KF643955, KF643956, KF644043, KF644059, KF644073, KF644107, KF644123, KF644190, KF644206, KF644232, KF644263, KF644321, KF644327	41	Layton et al., 2014
GQ902548-GQ902685	138	Marko et al., 2010
MN546832-MN546835, MN546842, MN546848, MN546852-MN546858	15	Yonemitsu et al., 2019
KM192133	1	Zbawicka et al., 2014
MN119673	1	Chung et al., 2019
MG422062	1	deWaard et al., 2019
MT736560-MT736683	124	Laakkonen et al., 2020
KF931763-KF931851, KF931854-KF931898, KF931901-KF931939, KF931943-KF932262	493	Crego-Prieto et al., 2015
MW191702-MW191719, MT857958-MT857963, MW150800-MW150801	28	This study

Table S2. The list of EF1α and CR sequences revealed by molecular cloning.

Locus	Individual	Tissue	Clone	Haplotype
EF1α	J17	Hemolymph	2	D
EF1α	J17	Hemolymph	3	D
EF1α	J17	Hemolymph	5	D
EF1α	J17	Hemolymph	8	D
EF1α	J17	Hemolymph	9	D'
EF1α	J17	Hemolymph	10	D'
EF1α	J17	Hemolymph	18	D'
EF1α	J17	Hemolymph	24	D'
EF1α	J17	Hemolymph	26	D'
EF1α	J17	Hemolymph	27	D'
EF1α	J17	Hemolymph	30	D'
EF1α	J17	Hemolymph	32	D
EF1α	J17	Hemolymph	33	D'
EF1α	J17	Hemolymph	34	D'
EF1α	J17	Hemolymph	35	D'
EF1α	J17	Hemolymph	36	D
EF1α	J38	Hemolymph	1	10
EF1α	J38	Hemolymph	2	10
EF1α	J38	Hemolymph	3	10
EF1α	J38	Hemolymph	4	10
EF1α	J38	Hemolymph	6	10
EF1α	J38	Hemolymph	9	10
EF1α	J38	Hemolymph	10	10
EF1α	J38	Hemolymph	11	10
EF1α	J38	Hemolymph	13	10
EF1α	J38	Hemolymph	15	10
EF1α	J38	Hemolymph	16	10
EF1α	J38	Hemolymph	17	10
EF1α	J38	Hemolymph	18	10
EF1α	J38	Hemolymph	19	10
EF1α	J38	Hemolymph	21	10
EF1α	J38	Hemolymph	27	10
EF1α	J54	Hemolymph	1	MINOR
EF1α	J54	Hemolymph	2	D
EF1α	J54	Hemolymph	5	MINOR
EF1α	J54	Hemolymph	6	H
EF1α	J54	Hemolymph	9	MINOR
EF1α	J54	Hemolymph	11	MINOR
EF1α	J54	Hemolymph	15	MINOR
EF1α	J54	Hemolymph	16	D
EF1α	J54	Hemolymph	17	D
EF1α	J54	Hemolymph	18	MINOR
EF1α	J54	Hemolymph	19	D
EF1α	J54	Hemolymph	20	D
EF1α	J54	Hemolymph	21	D
EF1α	J54	Hemolymph	24	MINOR
EF1α	J54	Hemolymph	26	D
EF1α	J54	Hemolymph	31	G1
EF1α	J54	Hemolymph	33	G
EF1α	J54	Hemolymph	41	1
EF1α	J54	Hemolymph	49	G

EF1α	J54	Foot	3	1
EF1α	J54	Foot	4	1
EF1α	J54	Foot	5	1
EF1α	J54	Foot	6	1
EF1α	J54	Foot	7	MINOR
EF1α	J54	Foot	8	1
EF1α	J54	Foot	9	D
EF1α	J54	Foot	10	3
EF1α	J54	Foot	11	3
EF1α	J54	Foot	12	1
EF1α	J54	Foot	13	1
EF1α	J54	Foot	14	1
EF1α	J54	Foot	15	3
EF1α	J54	Foot	19	1
EF1α	J54	Foot	17	1
EF1α	J54	Foot	18	MINOR
EF1α	J111	Hemolymph	2	G
EF1α	J111	Hemolymph	3	G
EF1α	J111	Hemolymph	7	G
EF1α	J111	Hemolymph	9	G
EF1α	J111	Hemolymph	11	MINOR
EF1α	J111	Hemolymph	12	5
EF1α	J111	Hemolymph	13	G
EF1α	J111	Hemolymph	14	H
EF1α	J111	Hemolymph	15	5
EF1α	J111	Hemolymph	17	H
EF1α	J111	Hemolymph	18	G
EF1α	J111	Hemolymph	19	4
EF1α	J111	Hemolymph	20	G
EF1α	J111	Hemolymph	24	G
EF1α	J111	Hemolymph	25	H
EF1α	J111	Hemolymph	27	G
EF1α	J111	Hemolymph	28	G
EF1α	J111	Hemolymph	29	2
EF1α	J111	Hemolymph	30	H
EF1α	J111	Hemolymph	31	G1
EF1α	J111	Hemolymph	32	2
EF1α	J111	Hemolymph	33	G
EF1α	J111	Hemolymph	34	MINOR
EF1α	J111	Hemolymph	35	4
EF1α	J111	Hemolymph	37	H
EF1α	J111	Foot	3	4
EF1α	J111	Foot	4	4
EF1α	J111	Foot	5	4
EF1α	J111	Foot	9	4
EF1α	J111	Foot	14	4
EF1α	J111	Foot	16	4
EF1α	J111	Foot	17	4
EF1α	J111	Foot	18	H
EF1α	J111	Foot	19	2
EF1α	J111	Foot	20	2
EF1α	J111	Foot	25	4
EF1α	J111	Foot	27	2
EF1α	J111	Foot	33	H
EF1α	J111	Foot	34	4

EF1α	J111	Foot	37	4
EF1α	J111	Foot	38	2
EF1α	J161	Hemolymph	1	H
EF1α	J161	Hemolymph	3	G
EF1α	J161	Hemolymph	6	D
EF1α	J161	Hemolymph	7	D
EF1α	J161	Hemolymph	9	G
EF1α	J161	Hemolymph	10	G
EF1α	J161	Hemolymph	13	H
EF1α	J161	Hemolymph	15	G
EF1α	J161	Hemolymph	18	MINOR
EF1α	J161	Hemolymph	20	G
EF1α	J161	Hemolymph	21	MINOR
EF1α	J161	Hemolymph	22	D
EF1α	J161	Hemolymph	23	G
EF1α	J161	Hemolymph	24	MINOR
EF1α	J161	Hemolymph	25	D
EF1α	J161	Hemolymph	26	D
EF1α	J161	Hemolymph	27	G
EF1α	J161	Hemolymph	28	MINOR
EF1α	J161	Hemolymph	29	MINOR
EF1α	J161	Hemolymph	30	H
EF1α	J161	Hemolymph	31	H
EF1α	J161	Hemolymph	33	MINOR
EF1α	J161	Hemolymph	34	D
EF1α	J161	Hemolymph	35	H
EF1α	J161	Foot	1	MINOR
EF1α	J161	Foot	2	MINOR
EF1α	J161	Foot	5	7
EF1α	J161	Foot	6	G
EF1α	J161	Foot	7	6
EF1α	J161	Foot	10	G
EF1α	J161	Foot	11	7
EF1α	J161	Foot	14	MINOR
EF1α	J161	Foot	18	7
EF1α	J161	Foot	20	H
EF1α	J161	Foot	21	6
EF1α	J161	Foot	23	6
EF1α	J161	Foot	28	6
EF1α	J161	Foot	33	7
EF1α	J161	Foot	35	MINOR
EF1α	J161	Foot	36	6
EF1α	J181	Hemolymph	1_pJet	G
EF1α	J181	Hemolymph	2_pJet	G
EF1α	J181	Hemolymph	3_pJet	G
EF1α	J181	Hemolymph	4_pJet	G
EF1α	J181	Hemolymph	5_pJet	G
EF1α	J181	Hemolymph	6_pJet	G
EF1α	J181	Hemolymph	7_pJet	G
EF1α	J181	Hemolymph	8_pJet	MINOR
EF1α	J181	Hemolymph	9_pJet	H
EF1α	J181	Hemolymph	10_pJet	G
EF1α	J181	Hemolymph	11_pJet	H
EF1α	J181	Hemolymph	12_pJet	G
EF1α	J181	Hemolymph	13_pJet	G

EF1α	J181	Hemolymph	14_pJet	G
EF1α	J181	Hemolymph	15_pJet	H
EF1α	J181	Hemolymph	16_pJet	MINOR
EF1α	J181	Hemolymph	1	MINOR
EF1α	J181	Hemolymph	2	G
EF1α	J181	Hemolymph	5	MINOR
EF1α	J181	Hemolymph	6	H
EF1α	J181	Hemolymph	7	H
EF1α	J181	Hemolymph	8	G
EF1α	J181	Hemolymph	9	H
EF1α	J181	Hemolymph	10	G
EF1α	J181	Hemolymph	12	G
EF1α	J181	Hemolymph	13	G
EF1α	J181	Hemolymph	15	G
EF1α	J181	Hemolymph	16	G
EF1α	J181	Hemolymph	18	H
EF1α	J181	Hemolymph	22	G
EF1α	J181	Hemolymph	23	H
EF1α	J181	Hemolymph	24	G
EF1α	J181	Hemolymph	25	H
EF1α	J181	Hemolymph	26	H
EF1α	J181	Hemolymph	28	H
EF1α	J181	Hemolymph	29	H
EF1α	J181	Hemolymph	30	MINOR
EF1α	J181	Hemolymph	31	G
EF1α	J181	Hemolymph	32	G
EF1α	J181	Hemolymph	33	G
EF1α	J181	Hemolymph	34	MINOR
EF1α	J181	Hemolymph	35	G
EF1α	J181	Hemolymph	36	H
EF1α	J181	Foot	2	8
EF1α	J181	Foot	4	8
EF1α	J181	Foot	6	8
EF1α	J181	Foot	16	8
EF1α	J181	Foot	17	9
EF1α	J181	Foot	20	9
EF1α	J181	Foot	23	8
EF1α	J181	Foot	24	9
EF1α	J181	Foot	28	8
EF1α	J181	Foot	29	8
EF1α	J181	Foot	30	8
EF1α	J181	Foot	31	8
EF1α	J181	Foot	39	9
EF1α	J181	Foot	47	8
EF1α	J181	Foot	51	8
EF1α	J181	Foot	58	8
CR	J54	Hemolymph	1	1
CR	J54	Hemolymph	2	1
CR	J54	Hemolymph	4	1
CR	J54	Hemolymph	5	1
CR	J54	Hemolymph	6	1
CR	J54	Hemolymph	7	1
CR	J54	Hemolymph	8	1
CR	J54	Hemolymph	10	1
CR	J54	Hemolymph	11	1

CR	J54	Hemolymph	12	1
CR	J54	Hemolymph	14	1
CR	J54	Hemolymph	15	1
CR	J54	Hemolymph	16	1
CR	J54	Hemolymph	19	1
CR	J54	Hemolymph	20	1
CR	J54	Hemolymph	21	1
CR	J111	Hemolymph	1	2
CR	J111	Hemolymph	3	2
CR	J111	Hemolymph	6	2
CR	J111	Hemolymph	12	2
CR	J111	Hemolymph	15	2
CR	J111	Hemolymph	2	4
CR	J111	Hemolymph	7	4
CR	J111	Hemolymph	10	4
CR	J111	Hemolymph	13	4
CR	J111	Hemolymph	14	4
CR	J111	Hemolymph	16	4
CR	J111	Hemolymph	8	MINOR
CR	J111	Hemolymph	4	MINOR
CR	J111	Hemolymph	11	MINOR
CR	J111	Hemolymph	17	MINOR
CR	J111	Hemolymph	5	MINOR
CR	J161	Hemolymph	1	1
CR	J161	Hemolymph	2	1'
CR	J161	Hemolymph	3	1'
CR	J161	Hemolymph	4	1
CR	J161	Hemolymph	5	1
CR	J161	Hemolymph	6	1
CR	J161	Hemolymph	7	1
CR	J161	Hemolymph	8	1
CR	J161	Hemolymph	9	1'
CR	J161	Hemolymph	10	1
CR	J161	Hemolymph	11	1'
CR	J161	Hemolymph	13	1
CR	J161	Hemolymph	14	1'
CR	J161	Hemolymph	15	1
CR	J161	Hemolymph	16	1
CR	J161	Hemolymph	17	MINOR
CR	J181	Hemolymph	1	2
CR	J181	Hemolymph	5	2
CR	J181	Hemolymph	6	2
CR	J181	Hemolymph	10	2
CR	J181	Hemolymph	11	2
CR	J181	Hemolymph	12	2
CR	J181	Hemolymph	15	2
CR	J181	Hemolymph	16	2
CR	J181	Hemolymph	17	2
CR	J181	Hemolymph	4	6
CR	J181	Hemolymph	7	6
CR	J181	Hemolymph	9	6
CR	J181	Hemolymph	2	MINOR
CR	J181	Hemolymph	13	MINOR
CR	J181	Hemolymph	20	MINOR
CR	J181	Hemolymph	14	MINOR

Table S3. NCBI accession numbers of cancer and host sequences generated in this study.

Sequence	Suggested origin	NCBI Accession Number
COI-1	Cancer	MT857958
COI-2	Cancer	MT857959
COI-3	Host	MT857960
COI-4	Host	MT857961
COI-5	Host	MT857962
COI-6	Host	MT857963
COI-7	Host	MW150800
COI-8	Host	MW150801
CR-1	Cancer	MT877229
CR-1'	Cancer	MT877228
CR-2	Cancer	MT877230
CR-3	Host	MT877231
CR-4	Host	MT877233
CR-5	Host	MT877232
CR-6	Host	MT877234
CR-7	Host	MW013817
CR-8	Host	MW013818
EF1α-1	Host	MW187821
EF1α-2	Host	MW187822
EF1α-3	Host	MW187823
EF1α-4	Host	MW187824
EF1α-5	Host	MW187825
EF1α-6	Host	MW187826
EF1α-7	Host	MW187827
EF1α-8	Host	MW187828
EF1α-9	Host	MW187829
EF1α-D	Host	MW187831
EF1α-D'	Host	MW187832
EF1α-G1	Cancer	MW187833
EF1α-H	Cancer	MW187834
EF1α-G	Cancer	MW187835
COI-R7	Host	MW191702
COI-R11	Host	MW191703
COI-R13	Host	MW191704
COI-R14	Host	MW191705
COI-J10	Host	MW191706
COI-J11	Host	MW191707
COI-J12	Host	MW191708
COI-J13	Host	MW191709
COI-J14	Host	MW191710
COI-J15	Host	MW191711
COI-J16	Host	MW191712
COI-J2	Host	MW191713
COI-J3	Host	MW191714
COI-J4	Host	MW191715
COI-J6	Host	MW191716
COI-J7	Host	MW191717
COI-J8	Host	MW191718
COI-J9	Host	MW191719
